# Two case reports and a literature review of hyperphosphatasia with intellectual disability syndrome 2 caused by a *PIGO* mutation

**DOI:** 10.3389/fped.2025.1667477

**Published:** 2025-10-15

**Authors:** Xinyi Wang, Jingya Zhao, Xiaoke Zhao, Le Ding, Min Zhu, Yang Li

**Affiliations:** ^1^ Department of Rehabilitation, Children’s Hospital of Nanjing Medical University, Nanjing, Jiangsu, China; ^2^ Department of Neurology, Children’s Hospital of Nanjing Medical University, Nanjing, Jiangsu, China

**Keywords:** PIGO gene, hyperphosphatasia with mental retardation syndrome2, global developmental delay, elevated alkaline phosphatase, gene mutation

## Abstract

**Objective:**

This study investigates the clinical features and genetic mutations associated with hyperphosphatasia with impaired intellectual development syndrome-2 (HPIDS2).

**Methods:**

A retrospective analysis was performed on two HPIDS2 cases treated at the Department of Rehabilitation, Nanjing Children's Hospital, from 2019 to 2023. Clinical features and genetic characteristics were summarized through a literature review.

**Results:**

Genetic testing showed compound heterozygous variations in the *PIGO* gene for both patients (Patient 1: c.[2612A>C];[2361dup]; Patient 2: c.[2510T>A];[693C>G]), with c.[2510T>A] and c.[693C>G] identified as novel mutations.

**Conclusion:**

Global developmental delay, with or without hyperphosphatemia, may indicate HPIDS2. The level of alkaline phosphatase elevation could reflect disease severity and prognosis. Our cases expand the known pathogenic variations in the *PIGO* gene and phenotypic spectrum of HPIDS2.

## 1 Background

Hyperphosphatasia with impaired intellectual development syndrome-2 (OMIM: #614749) is an autosomal recessive genetic disorder caused by pathogenic variants in the *PIGO* gene. This disorder was initially identified and reported by Krawitz et al. in 2012. The primary features of this syndrome encompass moderate to severe global developmental delay, facial dysmorphism, short distal phalanges (pertaining to fingers or toes), and elevated serum alkaline phosphatase levels (hyperphosphatasemia). Additional symptoms may include seizures, reduced muscle tone, genitourinary malformations, and anorectal malformations. This condition is also referred to as Mabry syndrome (Mabry et al., 1970) and is characterized by a defect in the synthesis of glycosylphosphatidylinositol (GPI), which is categorized as an inherited GPI deficiency disorder (IGD). The *PIGO* gene plays a crucial role in GPI biosynthesis. In this report, we present two cases of HPIDS2 resulting from *PIGO* gene mutations and review the relevant literature to enhance the awareness of this condition among clinicians.

## 2 Materials and methods

### 2.1 Study participants

Following the acquisition of informed consent, we collected pedigree information, clinical data, blood samples, and imaging examinations from the families involved. Approval for human subject research was obtained from the Ethics Committee of the Children's Hospital of Nanjing Medical University (202406009-1).

### 2.2 Whole exome sequencing

Venous blood samples of 2 ml were separately collected from the patient and their parents and placed in ethylenediaminetetraacetic acid (EDTA) anticoagulant peripheral venous tubes. Genomic DNA was extracted using the Blood Genomic Column Medium Extraction Kit (Kangwei Century). Whole-exome sequencing (WES) of the proband was performed by Beijing Quanpu Medical Laboratory. The IDT xGen® Exome Research Panel v1.0 and xGen Exome Research Panel v2.0 capture probes were utilized for liquid hybridization with gDNA library sequences. Targeted DNA fragments were enriched, and a whole-exome library was constructed. The captured library underwent high-throughput sequencing (PE150) using the Illumina NovaSeq 6000 platform. The resulting sequencing data were aligned to the Ensembl reference genome GRCh37/hg19 using Burrows-Wheeler Aligner (BWA) software. Single nucleotide polymorphisms (SNPs) and insertion-deletion (Indel) variants were analyzed using Genome Analysis Toolkit (GATK) software. Detected SNPs and Indels were subsequently filtered and selected based on sequencing depth and mutation quality to acquire high-quality, reliable mutations. The identified high-quality variants were annotated using in-house developed variant annotation software, correlating with major databases including dbSNP, 1000 Genomes, ExAC, ESP, as well as OMIM, HGMD, ClinVar, and others. Various protein structure prediction tools—such as Provean, SIFT, PolyPhen2-HVAR, PolyPhen2-HDIV, M-Cap, Revel, MutationTaster, and MaxEntScan splice site prediction software—were employed to analyze the pathogenicity of the variants. The pathogenicity analysis of variant sites was conducted according to the 2015 diagnostic guidelines of the American College of Medical Genetics and Genomics (ACMG) and the 2018 specifications for “Clinical Single Gene Genetic Testing Reports.” Finally, the target sequences of the patient and their parents were validated using Sanger sequencing.

## 3 Case presentation

### 3.1 Clinical findings

**Figure 1:**
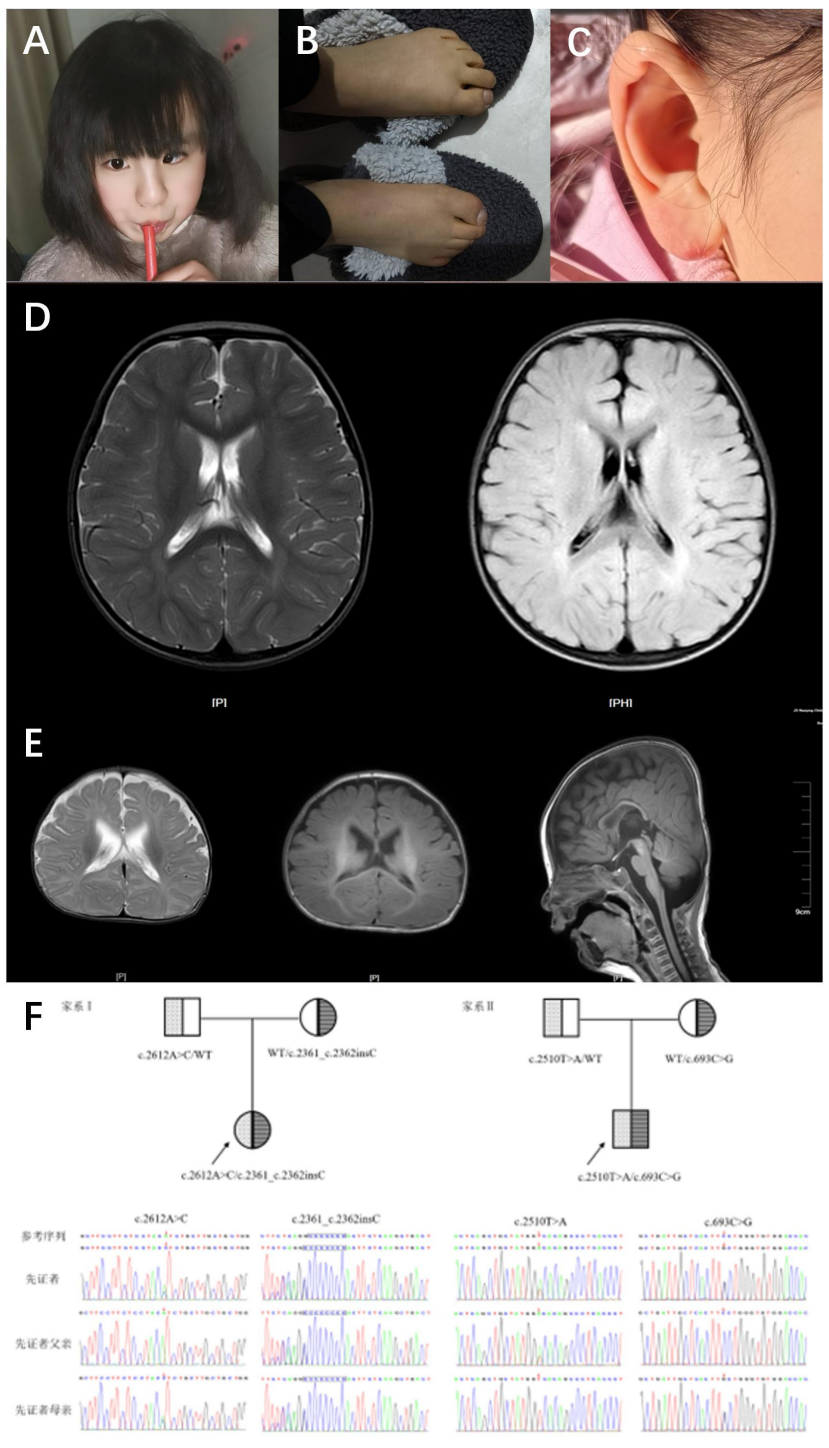
Typical features and brain MRI in 2 cases with HPIDS2. **(A)** Patient 1 (9 years 3 months) facial photo, showing left eye esotropia. **(B)** Patient 1 (9 years 3 months) foot and toe photo, showing thickened toes, yellow skin, and excessive skin keratinization. **(C)** Patient 1 (9 years 3 months) ear photo, showing ear malformation. **(D)** Patient 1's brain MRI shows the following findings: widened extra-axial spaces. **(E)** Patient 2's brain MRI shows the following findings: 1. Bilateral enlargement of the lateral ventricles, with widened extra-axial spaces in the temporal and frontal regions on both sides. 2. Slight delay in myelination. **(F)** Sanger sequencing chromatograms of PIGO gene mutations in two HPIDS2 patients from the family. The mutated bases are indicated by red arrows and blue boxes.

Patient 1 was a 5-year-and-2-month-old girl who presented to the Rehabilitation Department of Nanjing Children's Hospital in December 2019 due to “delayed language development.” During her initial consultation, she demonstrated the ability to call out names and produced approximately 10 words or syllables, including “ba,” “ma,” and “nai,” but was unable to form short sentences. She did not experience seizures but exhibited hyperactivity, poor concentration, and no signs of motor regression or stereotypic behaviors. The patient began walking independently at 24 months. The physical examination revealed decreased sensitivity in visual, auditory, and facial reflexes, along with ear deformities (Figure 1A), strong and bulky limbs, yellow skin color, and excessive skin keratinization (Figure 1B). Although the patient displayed a poor communicative attitude and did not cooperate during the examination, she could produce sounds spontaneously. She recognized facial features but could not indicate object size or quantity and could not distinguish colors; nevertheless, she could follow simple instructions. A brain MRI revealed widened extra-axial spaces. The parents reported that the patient's alkaline phosphatase levels fluctuated between 600 and 900 U/L during a follow-up phone call. To date, the patient has not received comprehensive rehabilitation treatment. Currently, she exhibits strabismus (Figure 1C) and has shown some improvement in both motor and cognitive abilities. She can ascend and descend stairs but cannot hop on one foot. There is increased muscle tone in both lower limbs, and she can now recognize colors and distinguish object sizes. She is able to follow simple instructions; however, there has been no significant improvement in her language skills. Her lexicon has expanded to approximately 30 words, including single words such as “ba”, “ma,” “nai,” “la,” and “yu,” but she remains unable to form short sentences. The patient is a full-term infant, classified as G1P1, delivered via Cesarean section with a birth weight of 3.5 kg. The pregnancy was uncomplicated, and the infant had APGAR scores of 10 at 1 and 5 min, with no history of asphyxia or hypoxia at birth. The infant experienced mild pathological jaundice with concurrent infection and has a history of recurrent otitis media. The parents are not closely related and deny any family history of hereditary neurological disorders.

Patient 2 was a 4-month-and-28-days-old boy who presented to the Rehabilitation Department of Nanjing Children's Hospital in August 2022 for “delayed motor and cognitive development.” Upon admission, he could not lift his head steadily, coughed during feeding, and exhibited upward eye deviation and limited spontaneous movement. EEG results were normal. The patient weighed 7 kg, had a triangular face with a high nasal bridge, and showed unstable muscle tone with involuntary movements. He could not bring both hands to the midline or grasp objects voluntarily. When pulled to a sitting position, he tilted his head back. In the prone position, he could not lift his head to 90 degrees or provide active elbow support, and when supported to sit, he leaned forward. Assisted standing revealed that his lower limbs could not bear weight. He could track objects to 90 degrees, responded to sounds, and exhibited nasal speech when crying. A prior MRI showed no significant abnormalities, and alkaline phosphatase levels were within normal limits. The patient's rehabilitation outcomes have been suboptimal; The patient has received intermittent comprehensive rehabilitation therapy to date, demonstrating improvement in motor skills and cognitive function compared to previous assessments. However, development remains delayed compared to age-matched peers. Currently, the patient is ambulatory but exhibits an abnormal gait characterized by pes valgus and external rotation of the feet. Communication skills are significantly impaired, with poor engagement and uncooperative responses during assessment. The patient is unable to follow simple instructions, differentiate sizes or quantities, or identify colors. Eye contact is fleeting, and vocalizations primarily consist of single syllables with an absence of words or phrases. Restricted interests are evident, manifested by repetitive lining up of toy cars. Cranial MRI reveals: 1. Bilateral lateral ventriculomegaly with widening of the extra-axial spaces in the temporal poles and frontal vertex. 2. Slightly delayed myelination. Elevated serum alkaline phosphatase (523 U/L) is also noted. The patient is classified as G2P2 (the first child is a healthy 7-year-old girl), born full-term via cesarean section with a birth weight of 3.7 kg, and the infant had APGAR scores of 10 at 1 and 5 min, with no history of asphyxia or hypoxia at birth. Mild jaundice was noted at birth. The mother experienced anemia during pregnancy, and parents are not closely related, denying any family history of hereditary neurological disorders.

### 3.2 Genetic analysis

**Figure 2:**
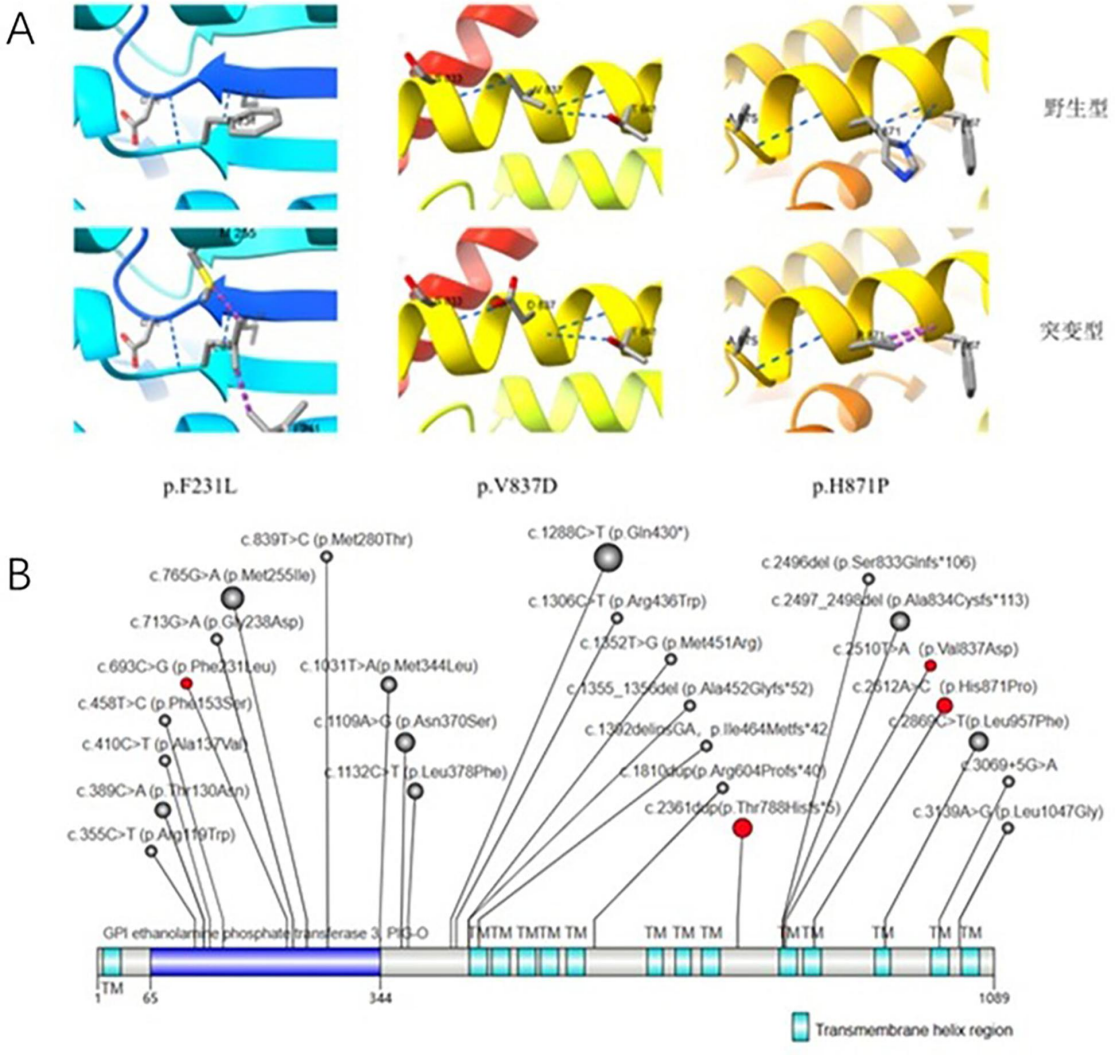
**(A)** Protein tertiary structure changes. Blue: Hydrogen bonds; Purple: Interacting forces (repulsion). **(B)** Illustrates reported *PIGO* gene mutations in HPIDS2 patients. The red background indicates mutations detected in the patients mentioned in this study. The size of the circles represents the frequency of detection (1–5).

Whole exome sequencing (WES) results revealed that Patient 1 and Patient 2 carried compound heterozygous variations in the *PIGO* gene: c.2612A>C (p.His871Pro)/c.2361dup (p.Thr788Hisfs*5) and c.2510T>A (p.Val837Asp)/c.693C>G (p.Phe231Leu), respectively (Figure 1F). According to the ACMG variant classification criteria, these variations were classified as variants of uncertain significance and pathogenic variants (Table 1). Multiple protein-damaging prediction software indicated deleterious effects, while tertiary structure simulations demonstrated alterations in hydrogen bonds and interacting forces, potentially impacting the correct folding and functional conformation of the active protein (Figure 2A). The phenotypes and genotypes of the proband and their parents exhibited co-segregation, consistent with the clinical phenotype and the autosomal recessive (AR) compound heterozygous inheritance mechanism. Therefore, the aforementioned genetic variants are believed to contribute to the pathogenesis of the disease.

**Table 1:** ACMG rating information.

Change of nucleotide	Change of amino acid	ACMG pathogenicity criteria	ACMG standard severity Level
c.2612A>C	p.His871Pro	PM1 + PM2_Supporting + PM3 + PP3 + PP4	Likely pathogenic
c.2361dup	p.Thr788Hisfs*5	PVS1 + PM2_Supporting + PP3 + PP4_Moderate	Pathogenic
c.2510T>A	p.Val837Asp	PM2_Supporting + PP3	Uncertain significance
c.693C>G	p.Phe231Leu	PM2_Supporting + PP3	Uncertain significance

**Table 2:** Genotypes and clinical manifestations in children with HPIDS2.

Patient's number Phenotype	In this report		Krawitz (2012)			Kuki (2013)	Nakamura (2014)		XUE (2016)	Zehavi (2017)		Pagnamenta (2017)	Tanigawa (2017)						Morren (2017)	Holtz (2020)	Tzovenos (2023)	Aguech (2023)
	Patient 1	Patient 2	1	2	3	4	5	6	7	8	9	10	11	12	13	14	15	16	17	18	19	20
Genotype	c.2612A>C(p.His871Pro)	c.2510T>A(p.Val837Asp)	c.2869C>T(p.Leu957Phe)	c.2869C>T(p.Leu957Phe)	c.2869C>T(p.Leu957Phe)	c.355C>T(p.Arg119Trp)	c.389C>A(p.Thr130Asn)	c.389C>A(p.Thr130Asn)	c.458T>C (p.Phe153Ser)	c.765G>A(p.Met255Ile)	c.765G>A(p.Met255Ile)	c.1306C>T(p.Arg436Trp)	c.1109A>G(p.Asn370Ser)	c.1109A>G(p.Asn370Ser)	c.1109A>G(p.Asn370Ser)	c.1031T>A(p.Met344Leu)	c.1031T>A(p.Met344Leu)	c.3139A>G(p.Leu1047Gly)	c.2612A>C(p.His871Pro)	c.1352T>G(p.Met451Arg)	c.410C>T(p.Ala137Val)	c.1132C>T(p.Leu378Phe)
	c.2361dup(p.Thr788Hisfs*5)	c.693C>G(p.Phe231Leu)	c.2361dup(p.Thr788Hisfs*5)	c.2361dup(p.Thr788Hisfs*5)	c.3069+5G>A	c.2497_2498del(p.Ala834Cysfs*113)	c.1288C>T(p.Gln430*)	c.1288C>T(p.Gln430*)	c.1355-1356del(p.Ala452Glyfs*52)	c.765G>A(p.Met255Ile)	c.765G>A(p.Met255Ile)	c.713G>A(p.Gly238Asp)	c.2497_2498del(p.Ala834Cysfs*113)	c.2497_2498del(p.Ala834Cysfs*113)	c.2496del(p.Ser833Glnfs*106)	c.1288C>T(p.Gln430*)	c.1288C>T(p.Gln430*)	c.1288C>T(p.Gln430*)	c.1810dup(p.Arg604Profs*40)	c.1392delinsGA(p.Ile464Metfs*42)	c.839T>C(p.Met280Thr)	c.1132C>T(p.Leu378Phe)
Gender	Female	Male	Female	Female	Female	Male	Male	Female	Male	Female	Female	Male	Female	Male	Female	Female	Female	Male	Male	Female	Female	Male
Nervous system																						
Global development delay	+	+	+	+	+	+	+	+	+	+	+	+	+	+	+	+	+	Language development delay	Intellectual disability	+	+	+
Microcephaly	NA	+	+	NA	+	NA	NA	NA	NA	NA	NA	+	NA	NA	NA	NA	NA	NA	NA	+	+	NA
Facial dysmorphism	Malformation of the ear screen, monocular squint	Upside-down triangle face, eyes rolled up	Widely spaced eyes, large eyes, long lid fissures, short nose, wide snout, tented mouth	Widely spaced eyes, large eyes, long lid fissures, short nose, wide snout, tented mouth	Widely spaced eyes, large eyes, long lid fissures, short nose, wide snout, tented mouth	Cleft lip and palate, excessive eye distance, blepharospasm	Highly arched palate, tented mouth	NA	cleft lip and palate	NA	Wide snout, low, distant ears, upward sloping lid fissures, blue sclera, long thin eyelashes	NA	Upward sloping eyelids, long lids, sparse hair, prominent nasal bridge, long and smooth manubrium, ear deformities, anteriorly tilted nostrils, cleft lip and palate	Upward sloping eyelids, long lids, sparse hair, prominent nasal bridge, long and smooth manubrium, ear deformities, anteriorly tilted nostrils, cleft lip and palate	Tented mouth, deformed ears, cleft lip and palate	Tented mouth, deformed ears	Tented mouth, deformed ears	High arched palate, cleft lip and palate, medial canthus, thick eyebrows	Nasal bridge broad, right ear anteriorly marked, upper lip w-shaped, mouth large, upper medial incisors fused, short neck	Upper oblique blepharoplasty, medial canthus crease, large and deformed ears, drooping corners of the mouth, tenting of the upper lip, small protruding palate	Large eyes, thin scalp, concave in front of the ears bilaterally, long philtrum	NA
Muscle tension	High	High	Low	Low	Low	Low	From low to high	Low	High	Low	Low	NA		Low	Low	Low	Low	Low	NA	NA	NA	NA
Epilepsy Seizures onset	NA	NA	NA	NA	21 months	1 years	1 years	7 months	6 months	10 months	6 months	NA	NA	NA	4 days	2 years	2 years	NA	17 months	2 months	NA	4 months
Sensorineural deafness	NA	NA	NA	NA	+	+	+	+	NA	NA	NA	+	+	+	+	NA	NA	NA	NA	+	+	NA
Chorea	NA	NA	NA	NA	NA	NA	+	NA	NA	NA	NA	NA	NA	NA	NA	NA	NA	NA	NA	NA	+	NA
Ataxia	NA	NA	NA	NA	NA	NA	NA	NA	NA	NA	NA	NA	NA	NA	NA	NA	NA	NA	NA	NA	NA	+
Brain MRI	Widened extra-axial spaces	1. bilateral enlargement of the lateral ventricles, widening of the temporal poles on both sides and the frontal parietal extracerebral space. 2. myelination is slightly behind.	NA	NA	Enlargement of the supratentorial ventricular system	Progressive cerebellar atrophy with abnormal high-intensity lesions of white matter throughout the brain	Diffuse cerebral and cerebellar atrophy	NA	Mild high signal on DWI around bilateral pallidum, dorsal brainstem and aqueducts	Hypoplasia of the corpus callosum and mild cortical atrophy	Severe hypoplasia of the corpus callosum/mild cortical atrophy, delayed myelin formation and hypoplasia of the corpus callosum	NA	NA	Cerebellar earthworm and brainstem hypoplasia	Atrophy of the cerebellar earthworms and reduction in the volume of cerebral white matter leading to ventricular dilatation	Normal	Normal	High signal in periventricular white matter, predominantly in the parieto-occipital lobes	Enlarged lateral ventricles, hyaline septum, thinning of the corpus callosum, small white matter lesions at the posterior horn	Thin corpus callosum, earthworm hypoplasia, optic nerve and optic crossings, prominent sulcus and cerebrospinal fluid spaces, and frontal-temporal subdural effusion	Left temporal lobe cyst, prominent fourth ventricle	NA
Integumentary system																						
Nail hypoplasia	NA	NA	+	+	+	+	NA	NA	NA	NA	NA	+	+	+	+	NA	NA	NA	+	+	+	NA
Hyperkeratotic skin	+	NA	NA	NA	NA	NA	NA	NA	NA	NA	NA	NA	NA	NA	NA	NA	NA	NA	+(with papillomatosis)	NA	NA	NA
Ichthyosis	NA	NA	NA	NA	NA	NA	NA	NA	NA	NA	NA	NA	+	+	+	NA	NA	NA	NA	NA	NA	NA
Skeletal system																						
Growth delay	NA	NA	+	NA	+	NA	+	+	NA	+	NA	NA	NA	NA	NA	NA	NA	NA	NA	NA	NA	NA
Short fingers/distal finger hypoplasia	NA	NA	+	+	+	+	NA	NA	+	NA	NA	NA	+	+	+	+	+	+	+	+	NA	NA
Stubby hands and feet	+	NA	NA	NA	NA	NA	NA	NA	NA	NA	NA	NA	NA	NA	NA	NA	NA	NA	NA	NA	NA	NA
Digestive system																						
Anal stenosis/atresia	NA	NA	+	+	+	NA	NA	NA	+	NA	NA	NA	+	+	NA	NA	NA	+	NA	+	NA	NA
Congenital megacolon	NA	NA	NA	NA	NA	+	NA	NA	NA	NA	NA	+	+	+	+	NA	NA	NA	NA	NA	+	NA
Feeding difficulty	NA	NA	NA	NA	NA	NA	NA	NA	NA	+	NA	NA	+	NA	+	NA	NA	NA	NA	+	NA	+
Urinary System	NA	NA	NA	NA	Vesicoureteral reflux	NA	NA	NA	NA	NA	NA	NA	NA	Rectourethral fistula	NA	NA	NA	NA	NA	Peripheral renal cyst	NA	NA
Reproductive System	NA	NA	NA	NA	Perineal fistula	NA	NA	NA	NA	NA	NA	NA	NA	Cryptorchidism	NA	NA	NA	Cryptorchidism	Hypogonadism	Uterine duplication	NA	NA
Circulatory System	NA	NA	NA	NA	Atrial septal defect	Fallot's tetralogy	NA	NA	NA	NA	NA	NA	NA	NA	NA	NA	NA	NA	NA	NA	NA	NA
Hyperphosphatasia(U/L)	+(600-900)	+(523)	+(1872)	+(1381)	+(1436)	+(1201-5959)	+(300-900)	NA	+(739–943)	+(217–241)	+(260–490)	+(418–624)	+(2816–5229)	+(2594)	+(3079)	+	+	–	+(5131)	+(> 1500)	+(929)	+(708)
Others	Recurrent otitis media	Trunk twisted, lots of involuntary movements	NA	NA	NA	NA	NA	NA	NA	NA	NA	NA	NA	NA	NA	NA	NA	NA	Papillomatosis, haemangioma	NA	NA	NA
Conclusion	NA	NA	NA	NA	Died of convulsive crisis at 22 months	NA	NA	Died of multi-organ failure at 1 year	NA	NA	NA	NA	Died of septicaemia at 17 months	Died of pneumonia at 18 months	NA	NA	NA	NA	NA	Died of infection at 9 months	NA	NA

“+” = present; “–” = absent; NA = unknown or not applicable.

## 4 Literature review

A literature search was conducted using the PubMed database with the keywords “HPIDS2” or “PIGO” from January 2001 to December 2023. The search focused on articles describing the clinical manifestations and genetic diagnosis of HPIDS2. A total of 11 relevant articles were identified, encompassing 20 patients. A summary of the clinical data and genetic testing results for these 20 cases, along with the two cases presented in this report, is provided in Table 2.

Through a literature review, we collected and analyzed the clinical data of 20 previously reported cases and 2 cases from this study (totaling 22 cases) of HPIDS2 patients. The results revealed the following prevalent clinical manifestations: intellectual developmental delay (21 cases), motor developmental delay (20 cases), craniofacial dysmorphism (18 cases), elevated serum alkaline phosphatase (ALP) levels (17 cases), abnormal brain magnetic resonance imaging (MRI) findings (14 cases), hypotonia (13 cases), distal phalangeal hypoplasia/aplasia (13 cases), epileptic seizures (12 cases), nail dysplasia/agenesis (11 cases), and sensorineural hearing loss (11 cases). Other less frequent features included microcephaly (5 cases) and hypertonia (2 cases). Furthermore, multi-system involvement was commonly observed: gastrointestinal abnormalities (15 cases), skeletal abnormalities (6 cases), cardiovascular abnormalities (2 cases) and genitourinary abnormalities (5 cases). Non-specific phenotypes included polyhydramnios (3 cases), hyperkeratosis (2 cases), vascular malformation (1 case), inguinal hernia (1 case), platelet dysfunction (1 case), and ataxia (1 case). Notably, six patients died in early childhood, primarily due to secondary infections and status epilepticus.

To date, all 22 patients with HPIDS2 have exhibited *PIGO* gene mutations, comprising a total of 26 mutations that include missense mutations (18), frameshift mutations (6), nonsense mutations (1), and non-coding region mutations (1). Among these, several mutations have been reported in multiple cases: c.1288C>T (p.Gln430*) (5 cases), c.2497_2498del (p.Ala834Cysfs113) (3 cases), c.2869C>T (p.Leu957Phe) (3 cases), c.1109A>G (p.Asn370Ser) (3 cases), and c.2361dup (p.Thr788Hisfs5) (3 cases), which are considered hotspots based on multiple reports (1–12).

## 5 Discussion

The *PIGO* gene is located on human chromosome 9p13.3, with its canonical transcript (NM_032634) comprising 10 protein-coding exons that encode a 1101-amino acid residue phosphatidylinositol glycan anchor biosynthesis class O protein (13). It encodes one catalytic component of GPI-EtNP transferase III, which attaches GPI to proteins by transferring ethanolamine phosphate (EtNP) to the third mannose (14). Over 100 mammalian cell surface proteins are tethered to the membrane by GPI attached at their C-termini. The PIGO protein plays a crucial role in the synthesis and maintenance of cellular membranes through its involvement in phosphatidylinositol glycosylation. Its functions include regulating membrane stability, participating in cell signaling, and modulating cell adhesion and migration (15).

Animal studies suggest that *PIGO* gene-deficient mice exhibit symptoms such as cognitive and motor impairment, delayed growth and development, and seizures, mirroring the clinical features of human HPIDS2. Additionally, *PIGO* gene deficiency in mice results in significant progressive brain structural changes: brain MRI shows reduced skeletal muscle volume, slightly decreased cerebellar volume, and a smaller pituitary gland, while brain volume remains unchanged. This differs from brain MRI findings in human HPIDS2, where some patients show abnormalities in corpus callosum development. Although these changes lack specificity, future identification of new cases should focus on neuroimaging changes (16). The neuroimaging findings in the cases of this study include reduced brain volume. Whether these changes are characteristic remains to be elucidated with more cases and through the discovery and summary of new variants. In conclusion, functional studies demonstrate the highly conserved nature of *PIGO* and its critical role in brain development, yet an explicit genotype-phenotype correlation has yet to be established.

We report two patients with HPIDS2, which was diagnosed clinically and genetically. These cases closely align with previously reported presentations of HPIDS2 patients. Both patients exhibited varying degrees of global developmental delay (Patient 1 primarily with delayed language development, and Patient 2 primarily with delayed motor and cognitive development). Additionally, both patients presented with craniofacial dysmorphisms (Patient 1 with ear deformity and Patient 2 with a triangular face and microcephaly). Mildly elevated serum alkaline phosphatase levels were observed in Patient 2, who additionally displayed bilateral upward gaze, trunk twisting, and involuntary movements. Furthermore, this study reported clinical manifestations not documented previously; Patient 1 exhibited monocular strabismus onset during school age, which may indicate an association between specific genotypes and ocular pathology, thus broadening the phenotypic spectrum of HPIDS2. Additionally, Patient 1 displayed excessive keratosis of the hands and feet, a feature also reported in patients with the p.His871Pro mutation by Morren in 2017. This study suggests a potential association between the p.His871Pro mutation and excessive skin keratosis, awaiting further evidence from additional cases (2). Furthermore, both patients exhibited increased muscle tension, contrasting with the predominantly decreased muscle tension reported in previous studies, indicating that decreased muscle tension is not a characteristic feature of HPIDS2.

In the initial report by Krawitz et al. in 2012, significantly elevated levels of alkaline phosphatase were observed in patients with HPIDS2 carrying *PIGO* mutations. Research indicates that abnormal GPI structure leads to signal peptide cleavage and soluble alkaline phosphatase secretion, causing elevated serum alkaline phosphatase in HPIDS2 patients (4). However, subsequent studies by Nakamura et al. in 2014, Xue et al. in 2016, and Zehavi et al. in 2017 indicated that significant elevation of alkaline phosphatase is not obligatory in HPIDS2 patients with *PIGO* mutations, consistent with the findings of this study. Neither of the two patients in this study exhibited severe deformities or seizures, and alkaline phosphatase levels were not significantly elevated (Patient 1 had normal or only mildly elevated alkaline phosphatase levels, while Patient 2 maintained normal alkaline phosphatase levels). Moreover, analysis of 20 previously reported cases revealed that children with significantly elevated alkaline phosphatase levels mostly presented with severe deformities and early-onset seizures. Cases of childhood mortality were primarily associated with markedly elevated alkaline phosphatase levels. Therefore, this study suggests a positive correlation between the degree of alkaline phosphatase elevation and disease severity, indicating that alkaline phosphatase levels can provide prognostic information for patients. In summary, based on the findings from these two cases, the clinical features of HPIDS2 patients can be succinctly summarized as “comprehensive developmental delay with or without hyperphosphatasemia,” providing convenience and potential for early detection of this disease for clinicians at all levels.

Currently, there is no specific treatment available for HPIDS2. The majority of affected children exhibit comprehensive developmental delay and decreased muscle tension. Symptomatic treatment and personalized comprehensive rehabilitation therapy, including physical therapy and cognitive training, aim to alleviate developmental delays as much as possible. For patients with seizures, oral vitamin B6 has shown some therapeutic effects, although the mechanism remains unclear and may be related to decreased expression of TNAP caused by *PIGO* mutations (17). Reports suggest that HPIDS2 patients are susceptible to seizure emergencies and infections, underscoring the importance of early seizure control and infection prevention. Additionally, evaluating alkaline phosphatase levels in patients can aid in determining disease severity and prognosis. Given the potential risk for genetic mutation transmission to offspring among affected individuals, genetic counseling during reproduction is advisable. Early diagnosis, intervention, and recognition of complications may effectively mitigate disease progression, improve the patient's quality of life, and enhance survival rates. Gene therapy is expected to be a promising direction in future research, involving assessment of cell surface GPI-AP activity to analyze feedback mechanisms and modulate GPI anchoring gene expression accordingly. The advent of gene editing technology represents a significant advancement in the potential treatment of rare genetic disorders. Successful case reports employing CRISPR-based (18) and customized lipid nanoparticle-delivered base-editing therapy (19) for delivery, have provided new hope and research pathways for addressing HPIDS2.

This study identified two previously unreported pathogenic variants associated with HPIDS2: c.2510T>A and c.693C>G. As previously mentioned, both affected children exhibited typical clinical manifestations of HPIDS2. Notably, one child presented with unilateral strabismus onset during school age. Furthermore, the p.His871Pro mutation may be associated with excessive skin keratinization, thereby expanding the spectrum of pathogenic variants in HPIDS2.

## Data Availability

All data generated or analysed during this study are included in this published article and its supplementary information files.
